# Influence of the Type of Breastfeeding and Human Milk Polyamines on Infant Anthropometric Parameters

**DOI:** 10.3389/fnut.2021.815477

**Published:** 2022-01-06

**Authors:** Nelly C. Muñoz-Esparza, Edgar M. Vásquez-Garibay, Elizabeth Guzmán-Mercado, Alfredo Larrosa-Haro, Oriol Comas-Basté, M. Luz Latorre-Moratalla, M. Teresa Veciana-Nogués, M. Carmen Vidal-Carou

**Affiliations:** ^1^Departament de Nutrició, Ciències de l'Alimentació i Gastronomia, Facultat de Farmàcia i Ciències de l'Alimentació, Campus de l'Alimentació de Torribera, Universitat de Barcelona, Santa Coloma de Gramenet, Spain; ^2^Institut de Recerca en Nutrició i Seguretat Alimentària (INSA·UB), Universitat de Barcelona, Santa Coloma de Gramenet, Spain; ^3^Xarxa d'Innovació Alimentària (XIA), Barcelona, Spain; ^4^Instituto de Nutrición Humana, Universidad de Guadalajara, Guadalajara, Mexico

**Keywords:** full breastfeeding, partial breastfeeding, infant growth, putrescine, polyamines, spermidine, spermine, adipogenesis

## Abstract

Feeding choices in the early months of life are key determinants of growth during infancy. Polyamines participate in cell proliferation and differentiation, and it has also been suggested that polyamine metabolism plays a role in adipogenesis. As the main exogenous source of polyamines in the infant is human milk, the aim of this work was to study if the type of breastfeeding received and the polyamine intake from human milk has an influence on infant anthropometric parameters. A cohort of 78 full-term healthy newborns was followed up until 4 months of age; 55 were fully and 23 partially breastfed. Anthropometric measurements were taken at 2 and 4 months, when human milk samples were also collected for analysis of polyamine content by UHPLC-FL. Fully breastfed infants had a better anthropometric profile than those partially breastfed (*p* < 0.05). Furthermore, polyamine intake in partially breastfed infants was significantly lower compared to those fully breastfed. However, only two of the 15 anthropometric indicators evaluated (triceps skinfold and mean upper arm circumference) showed a significant inverse association with polyamine content in human milk and intake (*p* < 0.05). Infant growth and body composition differ according to the type of breastfeeding received. Based on the weak associations between polyamines and anthropometric indicators, it is not possible to conclude the influence of polyamines in infant growth and body composition.

## Introduction

Besides determining infant growth, the feeding choices in the first months of life have a long-term health outcome, especially in the prevention of childhood obesity (1–3). It has been suggested that the protective effect of breastfeeding against the development of obesity could be related with the unique composition of human milk, which provides the energy and nutrients required by the infant, together with a range of bioactive compounds (e.g., hormones, immunoglobulins, growth factors, and polyamines) (4–6). It is well-established that infants who are exclusively breastfed have different patterns of growth and body composition compared to those who receive infant formulas (7–9); differences have also been found with infants that are partially breastfed, although this has been much less studied (2, 10, 11). Moreover, the specific mechanisms of the anti-obesity effect of breastfeeding remain unclear (3, 11).

The main exogenous source of polyamines for infants (i.e., putrescine, spermidine, and spermine) is human milk, in addition to *de novo* synthesis (12, 13). Polyamines are involved in several biological processes, mainly cell proliferation and differentiation, and protein synthesis; in the early stages of life, they also contribute to intestinal maturation and the development of the immune system (14, 15). More recently, it has been suggested that polyamine metabolism could also play a role in adipogenesis (16). The enzyme spermidine/spermine N1-acetyltransferase (SSAT) is a key metabolic regulator in polyamine homeostasis and is strongly involved in adipogenesis. SSAT catalyzes the transfer of acetyl groups from acetyl-CoA to spermidine or spermine, allowing polyamine interconversion (17, 18). Thus, the dysregulation of polyamine homeostasis could have an important impact on energy metabolism and the accumulation of body fat (16, 17). In fact, studies in mice report that overexpression of the SSAT enzyme caused a decrease in white adipose tissue (18, 19), and the administration of spermidine or spermine reduced body weight and fat mass, in a dose-dependent manner (20–22). However, as these results were obtained in animal models with induced obesity, they cannot be extrapolated to healthy infants.

It has been suggested that polyamine requirements are high in the first months of life due to the accelerated growth and development of the infant (the weight triples and length increases by 50%) (23, 24). However, to date, no studies have evaluated the role of human milk polyamines in infant growth and body composition. Bearing in mind the strong impact of early nutrition on development, as well as the importance of polyamines and their hypothetical involvement in adipogenesis, the aim of this work was to study whether the type of breastfeeding received and the polyamine intake from human milk has an influence on infant anthropometric parameters.

## Materials and Methods

### Study Design and Subjects

A non-randomized cohort study was conducted in healthy infants born at the Nuevo Hospital Civil de Guadalajara “Dr. Juan I. Menchaca” (Mexico) (25). The current study was carried out with a subgroup of 78 full-term healthy newborns, all with an appropriate weight for gestational age, until they were 4 months old. Among them, 55 infants were fully breastfed (optionally including oral hydration supplements and/or vitamins/inorganic nutrients) and 23 received partial breastfeeding (i.e., the infant received human milk and at least one bottle of infant formula/human milk substitutes). The inclusion and exclusion criteria, as well as the fieldwork strategy, are described in detail by Vásquez-Garibay et al. (25).

### Anthropometric Measurements

Anthropometric measurements of weight, length, head circumference, mean upper arm circumference (MUAC), triceps skinfold (TSF), and subscapular skinfold (SSF) were performed at 2 and 4 months of age according to the guidelines described by Frisancho (26). The techniques and instruments used to perform the aforementioned measurements are described by Vásquez-Garibay et al. (25). The *Z*-scores for weight/length, weight/age, length/age and body mass index (BMI)/age and the cephalic circumference, MUAC, TSF, and SSF were estimated using the software WHO Anthro 3.2.2 (WHO, Geneva, Switzerland).

### Analysis of Polyamines in Human Milk

Human milk samples were obtained from 9 am to 1 pm using a breast pump on the same day as infant anthropometric measurements were taken. Polyamine content was based on the mean values obtained in milk samples of ~5–10 mL corresponding to foremilk (milk available at the beginning of the feed) and hindmilk (milk at the end of the feed). After collection, the milk samples were stored at −80°C until the day of their analysis.

Sample preparation and chromatographic determination of polyamines in human milk were performed in the Food and Nutrition Campus of the University of Barcelona, according to the methods described in Muñoz-Esparza et al. (27) and Latorre-Moratalla et al. (28). Briefly, after sample acidification with perchloric acid, putrescine, spermidine, and spermine were separated and quantified by ion-pair ultra high-performance liquid chromatography coupled to an online derivatization with ortho-ophthaldehyde and subsequent fluorometric detection (UHPLC-FL).

### Estimation of Polyamine Intake

Infant polyamine intake was estimated considering a daily consumption of 800 ml of human milk according to the average infant human milk intake established by the Food and Agriculture Organization of the United Nations (29). For partially breastfed infants, a consumption of 50% human milk (400 ml) and 50% infant formula (400 ml) was standardized. The intake of polyamines in the latter scenario was estimated taking into account the polyamine content in milk of mothers practicing partial breastfeeding and the mean content of polyamines in infant formulas reported by Muñoz-Esparza et al. (30).

### Statistical Analysis

The statistical analysis of data was performed with the IBM SPSS Statistics 25.0 software package (IBM Corporation, Armonk, NY, USA). The non-parametric Mann-Whitney *U*-test was used to compare the two breastfeeding groups due to the lack of normal data distribution according to the Kolmogorov-Smirnov and Shapiro-Wilk tests. The Wilcoxon test was used to compare the polyamine content of human milk between 2 and 4 months. In addition, Spearman correlations were performed to evaluate the associations between polyamine contents in human milk and polyamine intake with the anthropometric parameters. The level of significance was a *p* ≤ 0.05.

## Results

Table 1 shows the anthropometric measurements and indices of infants at 2 and 4 months of age for the total cohort and according to the type of breastfeeding received. Compared to partially breastfed infants, those fully breastfed had significantly higher weight, BMI, MUAC, TSF, and SSF values, as well as higher *z*-scores for weight/age and weight/length, both at 2 and 4 months of age (*p* < 0.05).

**Table 1:** Anthropometric measurements and indices (Mean ± SD) of the infants at 2 and 4 months of age according to the type of breastfeeding.

**Anthropometric** **measurements and** **indices**	**2 months**				**4 months**			
	**Total cohort** **(***n*** = 78)**	**Full breastfeeding** **(***n*** = 55)**	**Partial breastfeeding** **(***n*** = 23)**	***p*** *	**Total cohort** **(***n*** = 78)**	**Full breastfeeding** **(***n*** = 55)**	**Partial breastfeeding** **(***n*** = 23)**	***p*** *
Weight (g)	5,309 ± 592	5,404 ± 617	5,084 ± 462	0.016	6,639 ± 727	6,708 ± 758	6,476 ± 632	0.071
Length (cm)	57.22 ± 1.64	57.22 ± 1.63	57.23 ± 1.69	0.367	62.34 ± 1.91	62.25 ± 2.00	62.57 ± 1.67	0.200
Weight/Age (z)	−0.35 ± 0.80	−0.22 ± 0.83	−0.65 ± 0.65	0.012	−0.30 ± 0.93	−0.21 ± 0.99	−0.52 ± 0.74	0.039
Length/Age (z)	−0.55 ± 0.78	−0.55 ± 0.76	−0.53 ± 0.86	0.447	−0.54 ± 0.81	−0.58 ± 0.86	−0.45 ± 0.69	0.225
Weight/Length (z)	0.26 ± 0.95	0.45 ± 0.90	−0.20 ± 0.90	0.004	0.11 ± 1.02	0.27 ± 1.05	−0.27 ± 0.87	0.017
BMI (kg/m^2^)	16.18 ± 1.36	16.47± 1.37	15.51 ± 1.10	0.002	17.06 ± 1.49	17.28 ± 1.53	16.53 ± 1.25	0.025
BMI (z)	−0.05 ± 0.90	0.13± 0.90	−0.50 ± 0.73	0.001	−0.01 ± 1.02	0.14 ± 1.06	−0.36 ± 0.85	0.020
Cephalic C. (cm)	38.70 ± 1.10	38.77 ± 1.17	38.56 ± 0.89	0.298	40.89 ± 1.12	40.89 ± 1.23	40.90 ± 0.88	0.357
Cephalic C. (z)	−0.26 ± 0.82	−0.24 ± 0.87	−0.35 ± 0.61	0.252	−0.38 ± 0.87	−0.38 ± 0.97	−0.38 ± 0.58	0.445
MUAC (cm)	11.96 ± 0.90	12.07 ± 0.97	11.71 ± 0.67	0.061	13.01 ± 0.93	13.11 ± 1.03	12.78 ± 0.59	0.056
MUAC (z)	n/a	n/a	n/a	–	−0.64 ± 0.89	−0.55 ± 0.99	−0.87 ± 0.55	0.029
TSF (mm)	8.62 ± 1.68	8.96 ± 1.77	7.83 ± 1.12	0.002	9.25 ± 1.85	9.46 ± 1.99	8.74 ± 1.39	0.040
TSF (z)	n/a	n/a	n/a	–	−0.29 ± 1.2	−0.17 ± 1.28	−0.57 ± 0.95	0.037
SSF (mm)	8.26 ± 1.68	8.61 ± 1.65	7.44 ± 1.50	0.002	8.33 ± 1.89	8.60 ± 1.95	7.69 ± 1.60	0.022
SSF (z)	n/a	n/a	n/a	–	0.42 ± 1.28	0.59 ± 1.31	0.01 ± 1.12	0.024

* *Significant differences between breastfeeding groups according to the Mann-Whitney U-test. (z), Z-score; BMI, Body Mass Index; MUAC, Mean Upper Arm Circumference; TSF, Triceps Skinfold; SSF, Subscapular Skinfold; Cephalic C, Cephalic Circumference; n/a, not applicable as it is not possible to estimate the Z-score*.

Polyamine contents in human milk were extremely variable among mothers, with relative standard deviations of over 54%, regardless of the polyamine and month (Table 2). Spermidine and spermine predominated in all samples, and were detected in very similar levels. Total polyamine, spermidine, and spermine contents were statistically lower at 4 vs. 2 months of breastfeeding (*p* < 0.05). Moreover, slightly lower values were found in the milk of mothers practicing full breastfeeding, although the differences were statistically significant only for putrescine and spermine at 2 months (*p* < 0.05).

**Table 2:** Polyamine content in human milk (nmol/dL) at 2 and 4 months in the total cohort and according to the type of breastfeeding (Mean ± SD, median, minimum, and maximum).

	**Total cohort** **(***n*** = 78)**	**Full breastfeeding** **(***n*** = 55)**	**Partial breastfeeding** **(***n*** = 23)**	***P*** *
**2 months**				
Total polyamines	668.37 ± 358.05 • 550.54 (213.37–1632.05)	639.98 ± 377.85 510.39 (213.37–1632.05)	737.50 ± 300.90 697.30 (265.71–1493.82)	0.088
Putrescine	41.89 ± 30.89 34.03 (3.40–147.48)	37.24 ± 32.28 22.69 (3.40–147.48)	53.02 ± 24.46 51.05 (17.02–113.44)	0.004
Spermidine	318.61 ± 170.21 275.39 (92.94–905.34)	312.33 ± 182.03 254.73 (92.94–905.34)	333.91 ± 139.77 309.81 (120.49–688.47)	0.257
Spermine	308.32 ± 177.97 252.05 (79.08–822.87)	290.97 ± 185.99 228.58 (79.08–822.87)	350.57 ± 152.22 333.60 (111.20–691.91)	0.050
**4 months**				
Total polyamines	554.70 ± 338.27 480.91 (136.31–1598.20)	524.85 ± 310.49 500.13 (136.31–1486.37)	632.05 ± 399.07 470.77 (156.80–1598.20)	0.431
Putrescine	38.15 ± 27.10 34.03 (1.13–153.15)	35.67 ± 26.14 28.36 (1.13–153.15)	44.58 ± 29.08 34.04 (5.11–119.12)	0.098
Spermidine	259.18 ± 154.47 234.08 (58.52–733.22)	252.08 ± 148.81 237.08 (58.52–733.22)	277.58 ± 170.54 218.59 (58.52–705.68)	0.678
Spermine	257.37 ± 178.89 217.46 (46.96–869.83)	237.09 ± 153.54 224.87 (46.96–743.80)	309.90 ± 228.03 212.52 (64.25–869.83)	0.299

*The Wilcoxon test was used to compare polyamine content at 2 and 4 months: p = 0.013 in total polyamines, p = 0.016 in spermidine, and p= 0.008 in spermine*.

* *Significant differences between breastfeeding groups according to the Mann-Whitney U-test*.

The estimated intake of total polyamines, spermidine, and spermine was up to 53% higher in fully vs. partially breastfed infants, both at 2 and 4 months (*p* < 0.05) (Table 3); in contrast, putrescine intake was 51 and 25% higher in partially breastfed infants at 2 and 4 months, respectively (*p* < 0.05).

**Table 3:** Estimation of polyamine intake of the infant according to the type of breastfeeding.

	**Total cohort** **(***n*** = 78)**	**Full breastfeeding^a^** **(***n*** = 55)**	**Partial breastfeeding^b^** **(***n*** = 23)**	***P*** *
**2 months**				
Total polyamines	4,554 ± 2743 3,797 (1,365–13,056)	5,080 ± 3,011 3,975 (1,707–13,056)	3,252 ± 1,204 3,091 (1,365–6,277)	0.016
Putrescine	319 ± 226 272 (27–1,180)	294 ± 258 182 (27–1,180)	379 ± 98 371 (235–621)	0.002
Spermidine	2,199 ± 1,343 1,790 (617–7,243)	2,499 ± 1,456 2,038 (744–7,243)	1,471 ± 559 1,374 (617–2,889)	0.003
Spermine	2,058 ± 1,358 1,700 (445–6,583)	2,328 ± 1,488 1,829 (633–6,583)	1,402 ± 609 1,334 (445–2,768)	0.008
**4 months**				
Total polyamines	3,774 ± 2,363 3,474 (302–11,891)	4,199 ± 2,484 4,001 (1,090–11,891)	2,720 ± 1,646 2,158 (302–6,695)	0.011
Putrescine	300 ± 188 272 (27–1,225)	286 ± 209 227 (27–1,225)	338 ± 120 303 (167–643)	0.024
Spermidine	1,781 ± 1,133 1,680 (135–5,866)	2,017 ± 1,191 1,900 (468–5,866)	1,197 ± 706 989 (135–2,958)	0.002
Spermine	1,714 ± 1,182 1,463 (257–5,950)	1,897 ± 1,228 1,799 (376–5,950)	1,240 ± 912 850 (257–3,479)	0.018

* *Significant differences between breastfeeding groups according to the Mann-Whitney U-test*.

a *Daily consumption of 800 ml of human milk*.

b *Daily consumption of 400 ml of human milk and 400 ml of infant formula*.

Only two of the fifteen evaluated anthropometric indicators were significantly correlated with the polyamine content in human milk at 2 and/or 4 months of age. Specifically, TSF (mm) showed a weak inverse correlation with putrescine (2 months: *r* = −0.322, *p* = 0.004; and 4 months: *r* = −0.246, *p* = 0.033); and spermine (2 months: *r* = −0.309, *p* = 0.006; and 4 months: *r* = −0.259, *p* = 0.025) concentrations, as did MUAC (cm) with spermidine (*r* = −0.302, *p* = 0.009) and spermine (*r* = −0.327, *p* = 0.004) at 4 months. After stratifying the total cohort according to the type of breastfeeding, these correlations were only found in the full breastfeeding group; TSF (mm) showed a weak inverse association with putrescine (*r* = −0.276, *p* = 0.043) at 2 months and with spermine (*r* = −0.311, *p* = 0.023) at 4 months, while MUAC was inversely correlated with putrescine (*r* = −0.380, *p* = 0.005) and spermine (*r* = −0.389, *p* = 0.004) at 4 months.

When Spearman's correlation test was run between anthropometric indicators and the estimated polyamine intake, weak inverse associations were again found only for the same two parameters. Thus, TSF (mm) showed a weak inverse correlation with putrescine intake at 2 (*r* = −0.361, *p* = 0.001) and 4 (*r* = −0.236, *p* = 0.04) months, as did MUAC (cm) with putrescine (*r* = −0.241, *p* = 0.036) and spermine (*r* = −0.253, *p* = 0.028) at 4 months. When stratifying the total cohort according to the type of breastfeeding, these correlations were only found in the full breastfeeding group. At 2 months, a weak inverse association was found between TSF (mm) and putrescine (*r* = −0.306, *p* = 0.023), and at 4 months between MUAC (cm) and putrescine (*r* = −0.291, *p* = 0.035) and spermine (*r* = −0.389, *p* = 0.004) and between TSF and spermine (*r* = −0.310, *p* = 0.024).

## Discussion

The worldwide prevalence of obesity has tripled in the last four decades and is now a serious public health concern (31). Children are not exempt from this epidemic, as according to the World Health Organization, 41 million under-fives were overweight or obese in 2016 (31). There is clearly an urgent need to improve strategies for the prevention and/or early detection of this health risk. Postnatal environmental factors, such as the type of feeding during the first months of life, have important effects on infant growth and body composition and influence long-term health outcomes, including childhood overweight and obesity (3, 10, 32). Patterns of infant development are known to differ greatly according to early nutrition (1, 9, 10, 33). For example, fully breastfed infants have a higher fat mass (both in grams and percentage) during the first 4 months of life, whereas formula-fed infants accumulate more lean mass and rapidly gain weight (1, 10, 32–34). Evidence supports that the accumulation of adipose tissue during breastfeeding is not associated with an increased risk of obesity in later stages of life (7, 9, 35, 36). Nevertheless, most of the available data are from studies comparing exclusively breastfed and formula-fed infants, with only a few including partially breastfed infants (2, 10, 11). In the present work, fully breastfed infants were found to have different anthropometric parameters in comparison with those partially breastfed, registering significantly higher weight, BMI, MUAC, TSF, and SSF, as well as greater *Z*-scores for weight/age and weight/length. These results are in agreement with Jia et al. (2), who also reported higher Z-scores for weight/age and weight/length in fully breastfed infants, and Rodríguez-Cano et al. (10), who found lower BMI, TSF, SSF, and fat mass (in kg and percentage) values in partially breastfed infants.

The variable content and distribution profile of polyamines found in human milk samples match previous reports by other authors (24, 37, 38). Moreover, the significant decrease in polyamine concentrations at 4 months is similar to that reported by Pollack et al. (39), Romain et al. (40), and Muñoz-Esparza et al. (30). The milk of mothers who partially breastfed had slightly higher polyamine contents than those fully breastfeeding, although this did not result in a greater polyamine intake by the infant. There are no data in the literature supporting the increased level of polyamines in milk from partially breastfeeding mothers. Nevertheless, it is likely that with longer periods between feeds, the milk in the mammary glands is more exposed to endo and exopeptidases, which could lead to an increase in free amino acids (polyamine precursors) (41, 42). According to our data, the polyamine intake in partially breastfed infants was 23–50% lower than in those fully breastfed, varying according to the individual polyamine and month of lactation, whereas the intake of putrescine was higher. These results could be attributed to the low total polyamine levels in infant formulas, described as up to 30-fold inferior compared to human milk (30), with the exception of putrescine, whose content is up to two-fold higher (24, 30). Overall, the potentially higher intake of polyamines in fully breastfed infants coincides with a higher growth and better anthropometric values than those found in partially breastfed infants.

Considering the high requirement for polyamines in the first months of life, a stage of accelerated growth and development, and the potential participation of polyamines in adipogenesis (16, 17), we studied if there was an association between the content of polyamines ingested through human milk and the infant anthropometric parameters. Most of the anthropometric indicators evaluated did not show significant correlations with the polyamine contents of human milk or polyamine intake, perhaps because all the infants in the study had an adequate nutritional status for their age, and anthropometric parameters were within normal limits (±2 SD). A significant relationship was only observed in two indicators, TSF and MUAC, which showed a weak inverse correlation with the content of putrescine and spermine in human milk and their intake, both in the total cohort and fully breastfed infants. It is important to bear in mind that other components of human milk, such as fat, carbohydrates, or proteins, could exert a greater influence on infant anthropometric parameters than polyamines. Thus, a limitation of this study is that the content of these nutrients in human milk was not evaluated. Further studies on the potential role of polyamines in infant growth and body composition should therefore measure other components of human milk, and include infants with abnormal anthropometric indicators. In addition, it would be desirable to apply more precise techniques for measuring body composition, such as DEXA (Dual Energy X-ray Absorptiometry) or ADP (Air displacement plethysmography). Another drawback of this study is the lack of information on the exact proportions of breast milk and infant formula consumed by the partially breastfed infants.

## Conclusion

The results of the current study confirm that infant growth and body composition differ according to the type of breastfeeding received. As expected, partially breastfed infants had a lower polyamine intake than those fully breastfed. However, a clear association could not be established between the levels of polyamines in human milk and most of the anthropometric indicators. Therefore, more studies are needed not only to confirm the potential role of polyamines in infant growth and body composition, but also to determine if a higher intake of polyamines through human milk results in higher blood polyamine concentrations in the infant.

## Data Availability Statement

The original contributions presented in the study are included in the article/supplementary material, further inquiries can be directed to the corresponding author.

## Ethics Statement

The studies involving human participants were reviewed and approved by Committees of Biosecurity, Bioethics and Research at the University of Guadalajara, Center of Health Sciences (CI-01314) and the Bioethics Commission of the University of Barcelona (IRB00003099). Written informed consent to participate in this study was provided by the participants' legal guardian/next of kin.

## Author Contributions

NM-E, EV-G, AL-H, ML-M, and MV-C: conceptualization and design of the study. NM-E, EG-M, EV-G, and AL-H were involved in the data collection. NM-E, ML-M, MV-N, and OC-B: analyzed the samples and interpreted the data. NM-E, EV-G, ML-M, MV-N, OC-B, and MV-C were involved in the writing original draft manuscript. NM-E, ML-M, MV-N, OC-B, and MV-C: critically reviewed the manuscript. All authors have read and approved the final version of the manuscript.

## Funding

This study received financial support from the National Council of Science and Technology of Mexico (234158). NM-E was a recipient of a doctoral fellowship from the University of Guadalajara, Mexico. This work was supported by Direcció General de Recerca (Generalitat de Catalunya, SGR-2017-1476).

## Conflict of Interest

The authors declare that the research was conducted in the absence of any commercial or financial relationships that could be construed as a potential conflict of interest.

## Publisher's Note

All claims expressed in this article are solely those of the authors and do not necessarily represent those of their affiliated organizations, or those of the publisher, the editors and the reviewers. Any product that may be evaluated in this article, or claim that may be made by its manufacturer, is not guaranteed or endorsed by the publisher.
